# Supplementary material for: The Vertebrate Breed Ontology: Toward Effective Breed Data Standardization
Source: J Vet Intern Med. 2025 May 25;39(4):e70133. doi: 10.1111/jvim.70133 (PMC12103836; doi:10.1111/jvim.70133)
Supplement: Supplementary file 1 — Data S1. [file JVIM-39-e70133-s001.pdf]

**Supplementary Table: Identifiers reported in this publication.**

| Categories | Labels | IDs | Location in the document |
|---|---|---|---|
| VBO term-breed | Aberdeen Angus, Brazil (Cattle) | VBO:0002150 | Fig.2 |
| VBO term-breed | Aberdeen Angus, Ireland (Cattle) | VBO:0002169 | Fig.2 |
| VBO term-breed | Aberdeen-Angus (Cattle) | VBO:0000090 | Fig.2 |
| VBO term-breed | American Miniature Horse (Horse) | VBO:0000896 | Text |
| VBO term-breed | Appaloosa (Horse) | VBO:0000904 | Text |
| VBO term-breed | Australian Mist (Cat) | VBO:0100034 | Text, Table 2 |
| VBO term-breed | Beagle (Dog) | VBO:0200131 | Text |
| VBO term-breed | Chihuahua (Dog) | VBO:0200338 | Table 1, Fig.1 |
| VBO term-breed | Chihuahua, Long-Haired (Dog) | VBO:0200339 | Text, Fig.1 |
| VBO term-breed | Chihuahua, Smooth-Haired (Dog) | VBO:0200340 | Text, Fig.1 |
| VBO term-breed | Cyprus (Cat) | VBO:0100081 | Text |
| VBO term-breed | Eastern Yak, Bhutan (Yak (domestic)) | VBO:0016815 | Fig.2 |
| VBO term-breed | Exotic Shorthair (Cat) | VBO:0100096 | Fig.3 |
| VBO term-breed | Foldex (Cat) | VBO:0100099 | Fig.3 |
| VBO term-breed | Guraghe, Ethiopia (Cattle) | VBO:0004734 | Fig.2 |
| VBO term-breed | Himalayan (Cat) | VBO:0100117 | Text, Fig.3 |
| VBO term-breed | Jersey Giant, Canada (Chicken) | VBO:0006068 | Table 1 |
| VBO term-breed | Knabstrupper (Horse) | VBO:0001008 | Text |
| VBO term-breed | Labradoodle (Dog) | VBO:0200798 | Text |
| VBO term-breed | Persian (Cat) | VBO:0100188 | Text, Fig.3 |
| VBO term-breed | Scandinavian Coldblood Trotter (Horse) | VBO:0017173 | Text |

| | | | |
|---|---|---|---|
| VBO term-breed | Siamese (Cat) | VBO:0100221 | Text, Fig.3 |
| VBO term-breed | Standardbred (Horse) | VBO:0000899 | Text |
| VBO term-breed | Zebu (Cattle) | VBO:0017417 | Fig.2 |
| VBO term-breed | Zebu, Australia (Cattle) | VBO:0004402 | Fig.2 |
| VBO term-breed | Zebu, Guyana (Cattle) | VBO:0004839 | Fig.2 |
| VBO term-classification | Alpaca breed | VBO:0000038 | Fig.1 |
| VBO term-classification | American bison breed | VBO:0000041 | Fig.1, Fig.2 |
| VBO term-classification | Amphibian breed | VBO:0400048 | Fig. 1 |
| VBO term-classification | Ass breed | VBO:0400005 | Fig.1 |
| VBO term-classification | Bird breed | VBO:0400006 | Text, Fig.1 |
| VBO term-classification | Bovine breed | VBO:0400019 | Fig.1, Fig.2 |
| VBO term-classification | Buffalo breed | VBO:0000068 | Fig.1, Fig.2 |
| VBO term-classification | Camel breed | VBO:0400022 | Fig.1 |
| VBO term-classification | Cat breed | VBO:0400018 | Fig.1, Fig.3 |
| VBO term-classification | Cattle breed | VBO:0400020 | Text, Fig.1, Fig.2 |
| VBO term-classification | Chicken breed | VBO:0400010 | Text, Fig. 4 |
| VBO term-classification | Deer breed | VBO:0400023 | Fig.1 |
| VBO term-classification | Dog breed | VBO:0400024 | Text, Fig.1 |
| VBO term-classification | Equid breed | VBO:0400033 | Fig.1 |
| VBO term-classification | Goat breed | VBO:0400025 | Fig.1 |
| VBO term-classification | Golden hamster breed | VBO:0400040 | Fig.1 |
| VBO term-classification | Guanaco breed | VBO:0000882 | Fig.1 |
| VBO term-classification | Guinea pig breed | VBO:0400026 | Fig.1 |
| VBO term-classification | Horse breed | VBO:0000931 | Fig.1 |

| | | | |
|---|---|---|---|
| VBO term-classification | Llama breed | VBO:0001098 | Fig.1 |
| VBO term-classification | Mammalian breed | VBO:0400049 | Fig. 1 |
| VBO term-classification | Partridge breed | VBO:0400038 | Text, Fig. 4 |
| VBO term-classification | Pheasant breed | VBO:0400037 | Text, Fig. 4 |
| VBO term-classification | Pig breed | VBO:0001199 | Fig.1 |
| VBO term-classification | Quail breed | VBO:0001223 | Text, Fig. 4 |
| VBO term-classification | Rabbit breed | VBO:0400029 | Fig.1 |
| VBO term-classification | South American camelid breed | VBO:0400032 | Fig.1 |
| VBO term-classification | Vertebrate Breed | VBO:0400000 | Text, Fig.1 |
| VBO term-classification | Vicuña breed | VBO:0001721 | Fig.1 |
| Taxon | *Bos* | NCBITaxon:9903 | Text, Fig.2 |
| Taxon | *Bos grunniens* | NCBITaxon:30521 | Fig.2 |
| Taxon | *Bos indicus* | NCBITaxon:9915 | Fig.2 |
| Taxon | *Bos indicus* × *Bos taurus* | NCBITaxon:30522 | Text |
| Taxon | *Bos taurus* | NCBITaxon:9913 | Fig.2 |
| Taxon | *Bovinae* | NCBITaxon:27592 | Fig.2 |
| Taxon | *Canis lupus familiaris* | NCBITaxon:9615 | Fig.1 |
| Taxon | *Coturnix* | NCBITaxon:9090 | Fig.4 |
| Taxon | *Gallus* | NCBITaxon:9030 | Fig.4 |
| Taxon | *Gallus gallus* | NCBITaxon:9031 | Text, Fig. 4 |
| Taxon | *Perdicinae* | NCBITaxon:466544 | Fig.4 |
| Taxon | *Phasianinae* | NCBITaxon:9072 | Text, Fig. 4 |
| Taxon | Vertebrata <vertebrates> | NCBITaxon:7742 | Fig.1 |
| Gene | PKD1, polycystin 1, transient receptor potential channel | NCBIGene:100144606 | Text |

| | | | |
|---|---|---|---|
| | interacting, Felis catus (domestic cat) | | |
| Disease | congenital stationary night blindness, TRPM1-related, horse | MONDO:1011255 | Text |
| Disease | polycystic kidney disease, domestic cat | MONDO:1011054 | Text |
| Breed status | domestication status | VBO:0300005 | Text |
| Breed status | extinction status | VBO:0300009 | Text |
| Breed status | fully recognized breed | VBO:0300002 | Text |
| Breed status | not recognized breed | VBO:0300004 | Text |
| Relation | *breed reported in geographic location* | VBO:0300020 | Text, Table 1 |
| Relation | *has foundation stock* | VBO:0300019 | Text, Table 1 |

Note that the category “VBO term-breed” includes sub-breed and variety.
